# Supplementary figures and images for: Using virtual reality hypnosis during stem cell transplant for patients in hematology: A protocol for a feasibility randomized study
Source: PLoS One. 2026 Feb 27;21(2):e0338617. doi: 10.1371/journal.pone.0338617 (PMC12948061; doi:10.1371/journal.pone.0338617)

**S1 Fig. Technical data sheets**

512


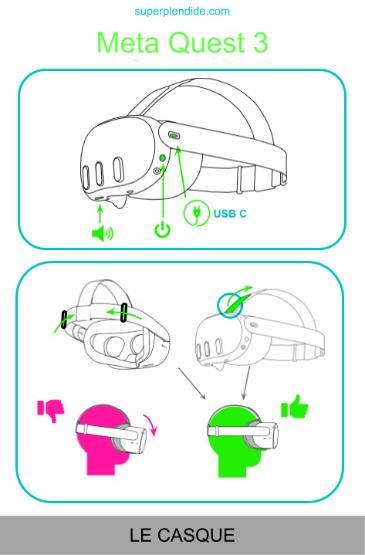

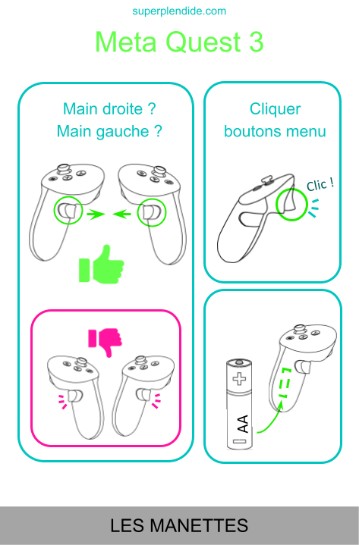

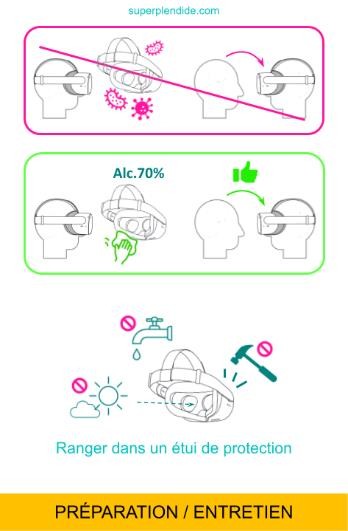


513


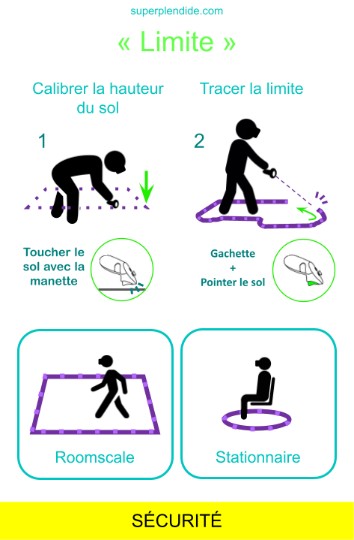

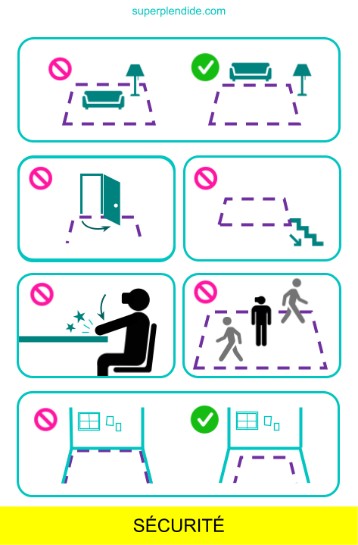

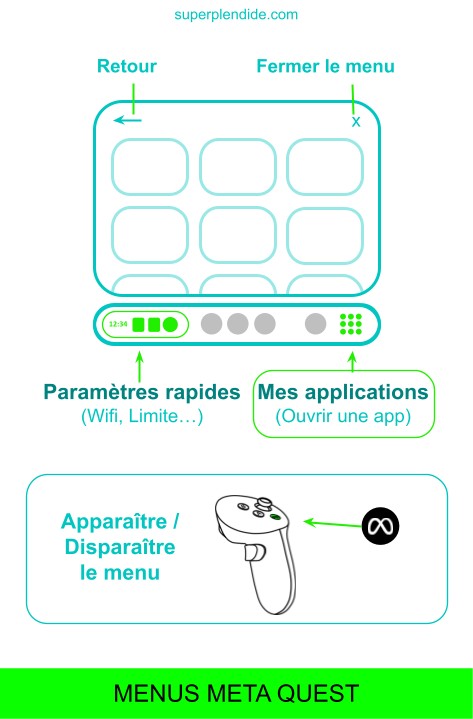


514


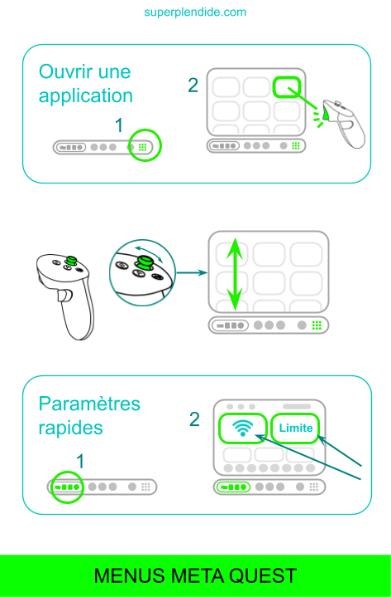

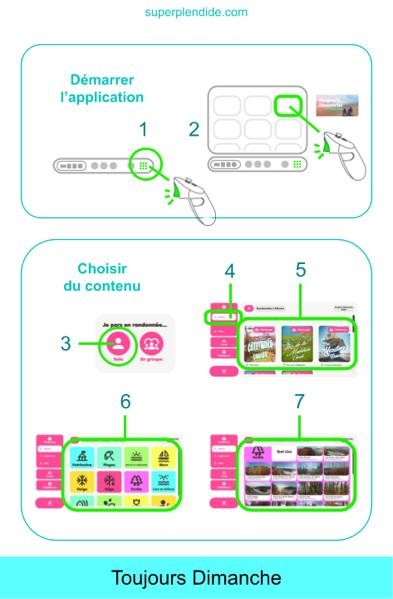

Supplement: S1 Fig — (DOCX) [file pone.0338617.s001.docx]
