# Supplementary material for: Using virtual reality hypnosis during stem cell transplant for patients in hematology: A protocol for a feasibility randomized study
Source: PLoS One. 2026 Feb 27;21(2):e0338617. doi: 10.1371/journal.pone.0338617 (PMC12948061; doi:10.1371/journal.pone.0338617)
Supplement: S3 File — (PDF) [file pone.0338617.s004.pdf]

Subscribe to DeepL Pro to edit this document.
Visit www.DeepL.com/pro for more information.

Development of an intervention integrating procedures combining hypnosis and virtual reality in the support of patients with myeloma and .lymphoma

PI: David Ogez, PhD, Clinical Assistant Professor

Co-PI: Richard Leblanc,

Study protocol

Maisonneuve-Rosemont Hospital / CIUSSS de l'est de l'Ile de Montréal

Amended of version 2024 -12-09

ClinicalTrials.gov ID: *TBD*

<u>Principal investigator :</u>

David Ogez, PhD, Psychologist,

Assistant Clinical Professor, Department of Anaesthesiology and Pain Medicine.

University of Montreal

Maisonneuve-Rosemont Hospital, CEMTL

5415, boulevard de l'Assomption

Montreal, Quebec

H1T 2M4 CANADA

+1-514-252-3400 Extension 4443

david.ogez@umontreal.ca

Nothing in [illegible]

permission of Dr. David Ogez.

Development and evaluation of an intervention integrating procedures combining hypnosis and virtual reality in the support of patients with myeloma and lymphoma.

Principal Investigator: David Ogez, PhD, Clinical Assistant Professor, Department of Anesthesiology and Pain Medicine, Université de Montréal

Co-Principal: Investigator Richard Leblanc, MD, Clinical Assistant Professor, Department of Anesthesiology and Pain Medicine, Université de Montréal

Co-investigators :

Philippe Richebé, MD, PhD, Full Professor, Department of Anesthesiology and Pain Medicine, Université de Montréal

Pierre Rainville, PhD, Professor, Faculty of Dentistry, Université de Montréal.

Karim Jerbi, PhD, Full Professor, Department of Psychology, Université de Montréal.

Mathieu Landry, PHD , MD, Department of Psychology, Université de Montréal.

Caroline Arbour, Associate Professor, Faculty of Nursing, Université de Montréal

Nadia Godin coordinator and research nurse

Moulay Idrissi MSc, BEng, Research Assistant

Floriane Rousseaux PhD, Department of Anesthesiology and Pain Medicine, Université de Montréal

Jade Véronneau, Research Assistant

Audrey Laurin, Msc, Department of Psychology, Université de Montréal, Maisonneuve-Rosemont Hospital

Valentyn Fournier, PhD, Postdoctoral Fellow, Department of Anesthesiology and Pain Medicine, Department of Medicine, Division of Hematology, Oncology and Transplantation, Maisonneuve-Rosemont Hospital, Université de Montréal

Patient partners: Danny Wade, Sandie Oberoi

Protocol summary

Title: **Development of an intervention integrating procedures combining hypnosis and virtual reality in the support of patients with myeloma and lymphoma**

Objective

Evaluate the effects of an RVH prototype in reducing anxiety and pain in transplant patients, and thus improving their long-term quality of life.

Gather user experience, satisfaction levels and recommendations for improvement of the RVH prototype from patients

Study drawing: Qualitative study of the RVH program user experience. And quantitative study to assess anxiety, pain, quality of life and fatigue.

Study population: haematology patients.

Sample size: 60 patients , 4 pre-test hematology patients

Study period: November 2024 - June 2025 .

Center responsible for the study: Centre de Recherche de l'Hôpital Maisonneuve-Rosemont (CR-HMR) - CEMTL, Montreal, Quebec, Canada.

Undesirable effects: none expected.

1. Issues

Quality of life and cancer

The measurement of quality of life is widely used in oncology. [1] It provides a vision of the patient's overall health in relation to the cancer diagnosis and treatments, and their physical (e.g., pain) and emotional (e.g., anxiety) impacts, anxiety). [2] Research with cancer patients has shown that quality of life is significantly altered by the disease and the treatments proposed to combat it. [3] Altered quality of life in these patients is also reported to be a predictor of significant emotional distress. [(4) . 5-7]

Multiple myeloma, lymphoma and stem cell transplantation

Multiple myeloma patients are among those most affected in terms of quality of life. [8] This blood cancer, which affects plasma cells, subtypes of white blood cells found in bone marrow, is the second most common hematological malignancy, and is among the top ten cancer diagnoses requiring hospitalization.[9] The cumulative time spent receiving treatment represents a significant burden for patients, and treatments have numerous adverse effects such as fatigue, nausea and weight loss.[10] These patients are faced with stem cell transplantation after high-dose chemotherapy, a treatment that is recognized as one of the most stressful in cancer therapy, due to numerous adverse effects, including pain, and the uncertainty linked to the fear of graft failure.[11] Studies have shown that the quality of life of these patients was greatly diminished during and immediately after autotransplantation, with a gradual improvement during the first year of follow-up. [8] Most of them regain a better quality of life three to five years after transplantation, while others risk developing depressive and anxiety disorders in the medium to long term. [8]

Studies report that 26% to 36% of patients with multiple myeloma report moderate to severe depressive symptoms in the first year after transplantation, and 18% of patients suffer from moderate to severe anxiety within the first 100 days after transplantation. [12,13] These rates are far higher than the general population, in which the prevalence is around 5% for depressive disorders, and 3% to 6% for generalized anxiety disorders.[14] In this context, patients with severely impaired quality of life and depression prior to stem cell transplantation are more likely to have impaired functional status after transplantation. [15-17] These data indicate a major interest in improving patients' quality of life during transplantation. By offering psychosocial management involving complementary techniques aimed at improving patients' quality of life during the sensitive period of transplantation, we can aim to reduce levels of emotional distress and thus prevent psychopathological complications in transplant patients.

Lymphoma is generally classified into two main types: non-Hodgkin's lymphoma (NHL) and Hodgkin's lymphoma. Both types of lymphoma are common indications for stem cell autotransplantation. Together, lymphoma and myeloma account for over 95% of all indications for autologous transplantation. The cumulative time spent receiving treatments represents a significant burden for patients, and treatments have numerous adverse effects such as fatigue, nausea and weight loss (Lewandowska et al., 2020). As a result, blood cancer patients are among the worst affected in terms of quality of life.

Virtual reality hypnosis

Psycho-oncology is a field of clinical intervention that has developed extensively over the last forty years. [18] Among other things, it advocates the importance of offering complementary intervention programs to better support oncology patients. [19] In contrast to the psychological interventions traditionally offered, complementary intervention programs can be more widely disseminated. They are more targeted, and aim, with certain benefits, at targets such as adaptation to cancer, treatment-related anxiety and patient pain. [19,20]

Among existing programs, technology-based interventions such as virtual reality combined with hypnosis (VRH) have shown conclusive results in the few randomized controlled trials published to date. [(21-23]VRH has also shown promising effects on pain and reductionstress .[23,24] A literature review on the usefulness of virtual reality (VR) during cancer-related medical procedures shows that it is adequate to distract from pain, even for very painful procedures, notably during chemotherapy.[25] Nevertheless, the authors deplore the lack of studies on HVR in oncology, the absence of multiple measures (self-reported, physiological) and, more generally, the ability of these studies to report on factors predicting the effective use of this technology (adherence, feasibility, user experience).[26] In addition, current HVR programs do not offer adaptability of the audio-visual environment in real time according to the patient's experience. [26] New paradigms need to be created to integrate care in a more individualized way. In a person-centered approach to care, it is essential to place the patient and his or her needs at the heart of the environment's design, by proposing a tool adapted to the care context and capable of delivering beneficial effects at the lowest cost. [25]

2. Objectives

The this main aim of is project to reduce anxiety and pain, and thus improve the quality of life of patients with hematological undergoing transplantationcancer The response we propose is in line with the recommendations issued by researchers in psycho-oncology and RVH, i.e., to develop an intervention program that can be widely disseminated and improve RVH protocols for better implementation.

For more than a year, clinicians, researchers, digital artists and patient partners have been working together to define an RVH program adapted to the problems of cancer, and usable by a large number of patients and caregivers. This program will have a number of advantages: it will enable individualized care through personalized hypnotic suggestions; it will enable the patient to be accompanied by the therapist through hypnosis verbatimspre-recorded ; it will promote better absorption of the patient into the RVH experience; and it will optimize response to hypnotic suggestions for managing emotions and pain.

This study will evaluate the user experience, satisfaction and recommendations of this HVR program in patients with hematological cancer undergoing stem cell transplantation, which will provide new guidelines for improving HVR applications, as well as assessing the prototype's effects on reducing anxiety and pain, and thus improving their long-term quality of life

Two complementary objectives were identified to evaluate the feasibility and effects of this program with a patient population highly affected by cancer, multiple myeloma, during a difficult treatment period, stem cell transplantation. In a first activity, we evaluated the user experience of this RVH program with patients in remission from cancer. This activity enabled us to define new guidelines for improving RVH applications. In the present project, we will evaluate the effects of RVH on quality of

life, patients' by modulating the negative emotions and pain experienced during stem cell transplantationanxiety and pain

3. Methodologies

RVH intervention

In collaboration with Super Splendide (www.supersplendide.com), a company specializing in the development of healthcare VR applications, our team, which includes two patient partners, has developed an intervention program (RVH) that combines VR and medical hypnosis (H) to facilitate the patient's absorption into a safe, benevolent imaginary world, in which they will be invited to live an experience that distracts them from their negative emotions and pain. The VR software was designed as part of another project developed in co-creation with palliative and long-term care settings (*Toujours dimanche:* https://vimeo.com/643281872). The development of *Toujours dimanche* involved over 200 volunteer hours of patient support, with the aim of adapting VR to the real needs of these patients. In the current project, the VRH program will offer a proactive approach to patient treatment. The virtual experience will be composed along the lines of a hypnosis session: 1- a soothing immersion will first be offered to anchor absorption , through calming audiovisual stimuli, 2- reaching a safe and relaxing space, representing a soothing natural environment (beach or park of the patient's choice), 3- two therapeutic stress management activities (putting all the stress you want to get rid of in a bag, and allow yourself to be carried by an imaginary horse, allowing you to escape to a safe place), 4- a physical discomfort modulation activity allowing you to reduce the sensation of discomfort in the part of your body afflicted by it, thanks to a visual representation of the pain that you extirpate from your own avatar and 5- return to an awake state of consciousness . The virtual environment increases the patient's sense of control, i.e. his ability to act and influence elements usually beyond his control, which in turn leads him to be more proactive in his therapy.

Evaluation of the RVH program user experience

Participants: A mixed study, combining quantitative and qualitative designs, was conducted with 40 partner patients in remission from cancer and 5 hospitalized hematology patients. For Activity 2, 60 participants will be recruited at clinicthe Maisonneuve-Rosemont Hospital oncology , after verification of eligibility in the medical records and with the attending physician, who will have received prior authorization from the patient to be contacted by the research team. Inclusion criteria for participants are: 18 years of age or older, French-speaking, being treated for lymphoma or myeloma and requiring a hematology transplant. Exclusion criteria are: deafness, blindness, confusion or other disorders that may impair communication.

Procedures Activity 1: As shown in figure 1, two sequences will be conducted. In the first sequence, partner patients in remission from cancer will be invited to experiment with and give feedback on the program in order to improve the RVH device, based on their personal experience. In the second phase, a pre-test of RVH will be carried out with patients hospitalized for a hematology transplant. These two sequences will consist of three stages. Participants will be invited to 1- use the RVH intervention in the company of a psychologist; 2- complete a questionnaire evaluating the user's experience; 3- take part in individual semi-structured interviews, conducted by a duo made up of a patient partner and a researcher, in order to gather explanations complementary to the scores attributed in the questionnaires.

Figure 1: Activity 1 protocol: User experience study

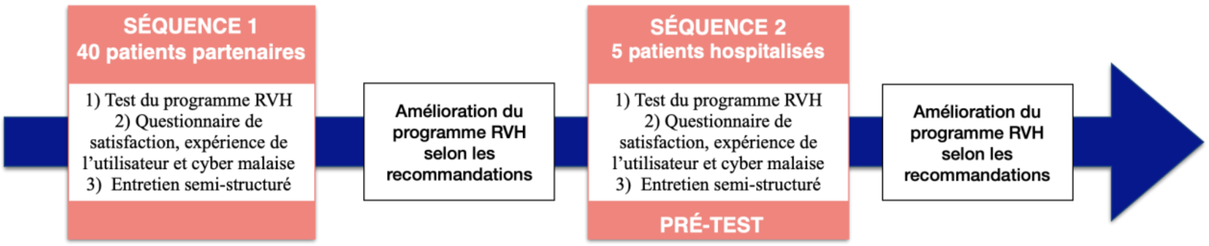

Procedures activity 2: As shown in figure 2, participants will be randomly assigned in a controlled manner with a ratio of 1:1 to two conditions: RVH and a control condition (C) consisting of usual care and a waiting list. This allocation will be done by a random draw using the *Research Randomizer* program (https://www.randomizer.org/), which will be conducted by a person independent of the study . The first meeting will take place on arrival at the hospital, where the study presentation will take place, and the consent form will be signed. Participants will be asked to complete self-assessments at three time points: T0=pre-, sessionT1=accompanied , and T2=post-transplantation (1 months) and at T3=follow-up (3 months) . According to studies carried out on multiple myeloma patients, T1 is conducted between D-14 and D-1 of the transplant stay.

Figure. Protocole : Étude des effets de l'intervention durant la transplantation de cellules souches

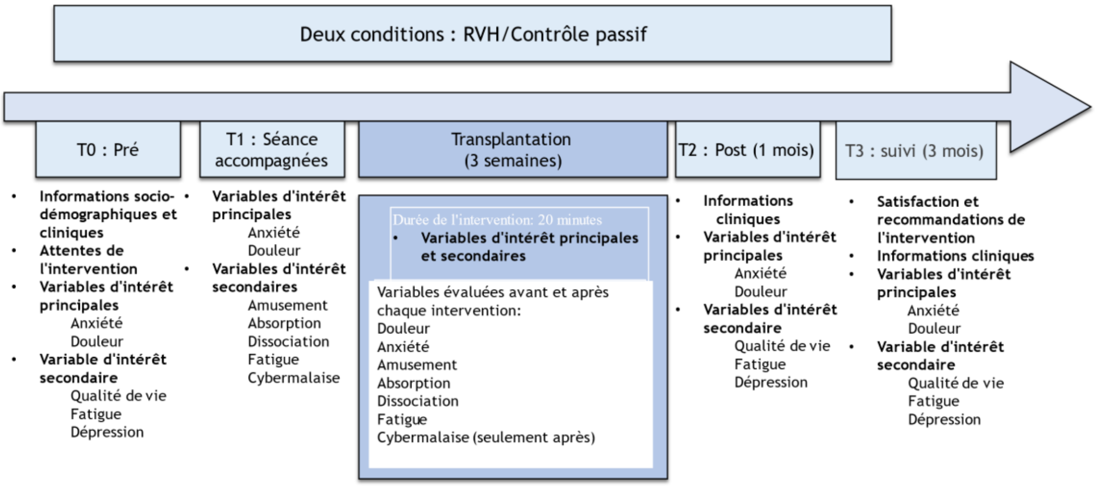

Measurements Activity 1: We will use two questionnaires. The first assesses the participant's satisfaction through a questionnaire19-item , which is evaluated on an Likert scale 11-level (0 = "strongly disagree" and 10 = "strongly agree" with the proposition). A questionnaire (UEQ, 24 items, 7-level Likert) will also be used to assess the six components of user experience: usability, usefulness, hedonic qualities (e.g., originality), attractiveness, sense of presence, resulting emotions. [27,28] The interview grid will be based on the same items as these questionnaires, enabling participants to develop their responses.

Measurements Activity 2: At T0, we will collect sociodemographic and data clinical from patients, including age, gender, sex, occupation, marital status, socioeconomic status and clinical diagnosis. We will also assess participants' expectations in the form of the question do you have any expectations regarding the use and efficacy of RVH? . At T0, T2 and T3 , we will collect data on quality of life, , anxiety , pain, fatigue and depression The FACT-BMT is a validated scale for patients undergoing bone marrow transplantation. It contains 50 items divided into 5 subscales: Physical Well-Being, Social/Family Well-Being, Emotional Well-Being, Functional Well-Being and Bone Marrow Transplant Subscale on a 5-point Likert scale ranging from 0 = not at all to 4 = very much. The *Hospital Anxiety Depression Scale* (HADS) is a scale validated in French for cancer, [34] which assesses participants' anxiety-depression. It is made up of 14 items: 7 items determine the degree of anxiety and the other 7, the degree of depression. The score of each subscale is calculated by summing the score of each item, which varies between 0 and 3, with each subscale reaching a maximum score of 21. [35] Fatigue will be assessed using the 10-item version of the Multidimensional Fatigue Inventory (MFI-10) adapted to cancer. It is scored on a 5-point Likert scale ranging from 1: "strongly disagree" to 5: "strongly agree". Pain is assessed using the Brief Pain Inventory (BPI). This scale consists of 8 items on a 10-point Lickert scale assessing pain severity, impact of pain on daily functions, location of pain, pain medication and extent of pain relief over the past 24 hours or week. At transplantation, we will use the diary with 11-level Scales before and after each RVH procedure to assess the direct effect of the procedure on pain, anxiety, enjoyment, i.-.i.e., the ability to enjoy oneself during the intervention, absorption, i.e., the tendency to become totally involved in a perceptual and imaginative experience, dissociation, i.e., a mental separation of components of the experience and fatigue. After each intervention, the Simulator Sickness Questionnaire, a scale validated in French, assesses symptoms of cybermalaise linked to immersion in VR. It contains 16 items divided into two subscales: nausea and oculomotor, which the participant must qualify as "not at all" (score 0), "a little" (score 1), "moderately" (score 2) or "severely" (score 3). . At T3 , we will collect patients' level of satisfaction through a 19-item questionnaire that is evaluated on an 11-point Likert scale (0 = "strongly disagree" and 10 = "strongly agree" with the proposal). This questionnaire has been used in other studies to assess participant satisfaction with an intervention program[37]. We will collect patients' levels of expectation and satisfaction using a five-level rating scale (1 = "not at all satisfied", 5 = "very satisfied") and explanatory interviews. Satisfaction interviews will also be conducted with caregivers in the hematology departments involved. The outcomesecondary will be the change from T0 to T2 and T3 in the scoresFACT-BMT, MFI and HADS-D, while the main outcomes primary will be changes from T0 in the degree of anxiety, of pain,. Changes in measures of pain, anxiety, amusement, absorption, and dissociation (logbooks completed during the two-week transplant stay) will also be considered secondary outcomes. We expect to At T3 , we will assess acceptability and user experience with the User Questionnaire Experience(UEQ). This validated questionnaire is composed of 24 items that are evaluated on a 7-level Likert scale. It will be used to assess the six components of user experience: usability, usefulness, hedonic qualities (e.g., originality), attractiveness, sense of presence, resulting emotions.[39,40] Sample size justification: At HMR, around 100 patients a year are treated for multiple myeloma and undergo cell transplantationstem The sample size was also determined using a power calculation to ensure a sufficient probability of detecting a statistically significant effect, if any. We used an alpha significance level of 0.05 and a power (1 - B) of 0.95. The expected effect was estimated from _____ , with an effect size of ______. Based on these

parameters, the power calculation suggests that a sample size of 60 participants is required to detect this effect with adequate power. The software ____ was used to perform this calculation. The sample size also takes into account a potential loss rate of ____%, to compensate for drop-outs and missing participants. .

Analyses Activity 1: The scores obtained on the questionnaire items will be compared with the theoretical mean of 6, i.e. the neutral level of acceptability on a scale from 0 to 10. An average above this threshold will be interpreted as a good experience of RVH. Analysis of the interview verbatim will enable us to make an open-ended assessment of the RVH experience. Significant narratives in each theme will be used to illustrate participants' opinions and experiences. Qualitative analysis of the narratives will enable us to identify positive and negative aspects, as well as avenues for improving the program. NVIVO software will be used for these analyses. This study will enable relevant modifications to be made to the program prior to the clinical effects study.

Analysis activity 2 :

Quantitative

In order to evaluate the efficacy of the intervention in the experimental group compared to the control group, a MANCOVA will be performed. The dependent variables are anxiety, depression, pain, quality of life and fatigue, and the independent variables are the different measurement times. We will control for the following : gender, age, socioeconomic status, diagnosis, absorption, dissociation and enjoyment. We will make post-hoc comparisons to compare scores between groups and at each measurement time. They will be made with a correctioncovariatesBonferroni .

Correlations will be performed on variables measured during the transplant phase, to measure the immediate effects of RVH.

Qualitative

The verbatims from the semi-structured interviews will be analyzed qualitatively using softwareNVivo . The software enables thematic content analysis, highlighting codes in the text and graphically grouping them into themes to create a thematic tree.

4. Benefits and expected results

Expected results include the development and refinement of an RVH program in collaboration with clinicians, researchers, digital artists and patient partners. Members of our team (DO, PR, FR) have developed considerable expertise in this area, notably through studies on the effects of hypnosis,[43-37] its effectiveness with cancer patients,[44-45] and the design of RVH studies.[21-46] The project also follows the development of a VR program by Super Splendid (JFM) to support palliative care patients,[47] and a self-hypnosis (DO training)developed with cancer patients.[44] The involvement of partner patients (DW, SO) is also at the heart of this project. Involved at all stages of the research, patient partners and patient groups will bring their personal experience to bear, greatly enhancing the feasibility of the program, and hence its implementation in cancer clinics. Feasibility is also supported by the expertise of clinicians (RL, CD, AGA). HMR is a referral center for multiple myeloma in Canada, so we know this patient population well. Testing a program with such a sensitive population brings with it many challenges, such as managing significant side effects associated with treatments that could make it difficult to use VR, taking into account patients' immunosuppression and preventing infections. We have anticipated these challenges in the development of the program, in conjunction with advice from hematologists and patient partners and the ergonomic expertise of our collaborators, using, for example, VR hardware

specific to this hospital context, easy-to-use software interfaces and headset disinfection kits. All the data gathered in this project will be of prime importance in promoting this program with wider populations of oncology patients.

Important research prospects are also identified as a result of this project. Our research team includes several researchers in human neuroscience and physiological measurement (PR, KJ, PhR, ML) whose expertise is essential for studies complementary to our project. These studies will incorporate physiological measurements non-invasiveof autonomic and brain activity to characterize responses to the intervention. These researchers are also involved in the development and evaluation of the intervention in a clinical context, to ensure that the experimental research protocols developed to assess neurophysiological mechanisms take account of the context of clinical applications. We anticipate that relevant complementary physiological measures will be incorporated into subsequent clinical studies

Following on from this project, we are planning multi-center efficacy studies with a wider population of cancer patients. To facilitate the opening up of this project to all cancer patients, an RVH user manual and a training program for healthcare staff are currently being developed, thanks to our collaborators: expert hypnosis training psychologists from Quebec, as well as collaborators from the University of Liège (ULG, Belgium) and Lille (France), involved in the tool development process thanks to their expertise in oncology hypnosis and RVH in clinical settings. All these steps will facilitate a multicenter randomized study with our collaborators (HMR, Sacré-Cœur, ULG, Université de Lille). The long-term objective is to propose the most effective, appropriate and comprehensive program for oncology patients, so that it can be used by a wide range of professionals (nurses, psychologists, educators, partners at the patient's bedside) and to extend this project to various centers cancer in the French-speaking world (Quebec, Belgium, France), in order to greatly improve ' quality of life. our patients

References :

1. Aaronson NK, Ahmedzai S, Bergman B, et al. The European Organization for Research and Treatment of Cancer QLQ-C30: a quality-of-life instrument for use in international clinical trials in oncology. J Natl Cancer Inst. 1993;85(5):365-376.

2. Mokhatri-Hesari P, Montazeri A. Health-related quality of life in breast cancer patients: review of reviews from 2008 to 2018. Health Qual Life Outcomes. 2020;18(1):338.

3. Jitender S, Mahajan R, Rathore V, Choudhary R. Quality of life of cancer patients. J Exp Ther Oncol. 2018;12(3):217-221.

4. Felder-Puig R, di Gallo A, Waldenmair M, et al. Health-related quality of life of pediatric patients receiving allogeneic stem cell or bone marrow transplantation: results of a longitudinal, multi-center study. Bone Marrow Transplant. 2006;38(2):119-126.

5. Ogez D, Colmant M, Zech E, de Timary P. When Psychologist-Patient Interactions are Systematic: Is there still room for genuinely personal support? Experiences among Cancer Patients. Psycho-oncology. 2014;8(2).

6. Benfer N, Bardeen JR, Fergus TA. The Interactive Effect of Attention to Emotions and Emotional Distress Intolerance on Anxiety and Depression. J Cogn Psychother. 2017;31(2):91-100.

7. Chin H, CG. N, Thong K, Seed H, Aili H. Demoralization in Cancer Patients: The Association with Distress, Depression and Positive Emotion. Med & Health. 2021;16(1): 108-122.

8. Martino M, Rossi M, Ferreri A, et al. Quality of life outcomes in multiple myeloma patients: a summary of recent clinical trials. Expert Rev Hematol. 2019;12(8):665-684.

9. van de Donk N, Pawlyn C, Yong KL. Multiple myeloma. Lancet. 2021;397(10272):410-427.

10. Rajkumar SV. Multiple myeloma: Every year a new standard? Hematol Oncol. 2019;37 Suppl 1:62-65.

11. Braamse AM, van Meijel B, Visser O, et al. Distress and quality of life after autologous stem cell transplantation: a randomized clinical trial to evaluate the outcome of a web-based stepped care intervention. BMC Cancer. 2010;10:361.

12. Chang G, Orav EJ, McNamara TK, Tong MY, Antin JH. Psychosocial function after hematopoietic stem cell transplantation. Psychosomatics. 2005;46(1):34-40.

13. Lee SJ, Loberiza FR, Antin JH, et al. Routine screening for psychosocial distress following hematopoietic stem cell transplantation. Bone Marrow Transplant. 2005;35(1):77-83.

14. WHO. Depression. https://www.who.int/fr/news-room/fact-sheets/detail/depression. Published 2021. Accessed 2 february 2022.

15. Syrjala KL, Langer SL, Abrams JR, et al. Recovery and long-term function after hematopoietic cell transplantation for leukemia or lymphoma. JAMA. 2004;291(19):2335-2343.

16. Andorsky DJ, Loberiza FR, Lee SJ. Pre-transplantation physical and mental functioning is strongly associated with self-reported recovery from stem cell transplantation. Bone Marrow Transplant. 2006;37(9):889-895.

17. Goetzmann L, Klaghofer R, Wagner-Huber R, et al. Psychosocial vulnerability predicts psychosocial outcome after an organ transplant: results of a prospective study with lung, liver, and bone-marrow patients. J Psychosom Res. 2007;62(1):93-100.

18. Holland JC. Psycho-oncology: Overview, obstacles and opportunities. Psychooncology. 2018;27(5):1364-1376.

19. Teo I, Krishnan A, Lee GL. Psychosocial interventions for advanced cancer patients: A systematic review. Psychooncology. 2019;28(7):1394-1407.

20. Pike A, Hearn L, de CWAC. Effectiveness of psychological interventions for chronic pain on health care use and work absence: systematic review and meta-analysis. Pain. 2016;157(4):777-785.

21. Rousseaux F, Bicego A, Ledoux D, et al. Hypnosis associated with 3d immersive virtual reality technology in the management of pain: A review of the literature. Journal of Pain Research. 2020;13:1129-1138.

22. Patterson DR, Jensen MP, Wiechman SA, Sharar SR. Virtual reality hypnosis for pain associated with recovery from physical trauma. Int J Clin Exp Hypn. 2010;58(3):288-300.

23. Patterson DR, Soltani M, Teeley A, Morse D, Wiechman SA, Gibran NS. Hypnosis delivered through immersive virtual reality for wound care: a randomized, controlled study. Journal of burn care & research. 2012;33(2):S70-.

24. Ong TL, Ruppert MM, Akbar M, et al. Improving the Intensive Care Patient Experience With Virtual Reality-A Feasibility Study. Crit Care Explor. 2020;2(6):e0122.

25. Indovina P, Barone D, Gallo L, Chirico A, De Pietro G, Giordano A. Virtual Reality as a Distraction Intervention to Relieve Pain and Distress During Medical Procedures: A Comprehensive Literature Review. Clin J Pain. 2018;34(9):858-877.

26. Fussell SG, Truong D. Using virtual reality for dynamic learning: an extended technology acceptance model. Virtual Real. 2021:1-19.

27. Loup-Escande E, Lécuyer A. Towards a user-centred methodological framework for the design and evaluation of applications combining brain-computer interfaces and virtual environments: contributions of ergonomics. In: INRIA R, ed2014.

28. Chaniaud N, Megalakaki O, Capo S, Loup-Escande E. Effects of User Characteristics on the Usability of a Home-Connected Medical Device (Smart Angel) for Ambulatory Monitoring: Usability Study. JMIR Hum Factors. 2021;8(1):e24846.

29. Gonsalves WI, Gertz MA, Dispenzieri A, et al. Implications of continued response after autologous stem cell transplantation for multiple myeloma. Blood. 2013;122(10):1746-1749.

30. Hickman KE. The Elkins Hypnotizability Scale: Validity, reliability, factor structure, and acceptability within a clinical sample. Dissertation Abstracts International: Section B: The Sciences and Engineering. 2019;80(1-B(E)):No Pagination Specified.

31. Nolte S, Liegl G, Petersen MA, et al. General population normative data for the EORTC QLQ-C30 health-related quality of life questionnaire based on 15,386 persons across 13 European countries, Canada and the United States. Eur J Cancer. 2019;107:153-163.

32. Bultz BD, Carlson LE. Emotional distress: the sixth vital sign--future directions in cancer care. Psycho-oncology. 2006;15(2):93-95.

33. Mitchell AJ. Pooled results from 38 analyses of the accuracy of distress thermometer and other ultra-short methods of detecting cancer-related mood disorders. Journal of clinical oncology : official journal of the American Society of Clinical Oncology. 2007;25(29):4670-4681.

34. Razavi D, Delvaux N, Farvacques C. Validation of the French version of the HADS in a population of hospitalized cancer patients. Revue de Psychologie Appliquée. 1989;39.

35. Annunziata MA, Muzzatti B, Bidoli E, et al. Hospital Anxiety and Depression Scale (HADS) accuracy in cancer patients. Support Care Cancer. 2020;28(8):3921-3926.

36. Wallston, K. A. (2005). The validity of the multidimensional health locus of control scales. Journal of health psychology, 10(5), 623-631.

37. Ogez D, Aramideh J, Mizrahi T, et al. Does practising hypnosis-derived communication techniques by oncology nurses translate into reduced pain and distress in their patients? An exploratory study. Br J Pain. 2021;15(2):147-154.

38. Rousseaux F, Dardenne N, Massion PB, et al. Virtual reality and hypnosis for anxiety and pain management in intensive care units: A prospective randomised trial among cardiac surgery patients. Eur J Anaesthesiol. 2022;39(1):58-66.

39. Pike A, Hearn L, de CWAC. Effectiveness of psychological interventions for chronic pain on health care use and work absence: systematic review and meta-analysis. Pain. 2016;157(4):777-785.

40. Rousseaux F, Bicego A, Ledoux D, et al. Hypnosis associated with 3d immersive virtual reality technology in the management of pain: A review of the literature. Journal of Pain Research. 2020;13:1129-1138.

41. Frodin U, Borjeson S, Lyth J, Lotfi K. A prospective evaluation of patients' health-related quality of life during auto-SCT: a 3-year follow-up. Bone Marrow Transplant. 2011;46(10):1345-1352.

42. Nielsen LK, Jarden M, Andersen CL, Frederiksen H, Abildgaard N. A systematic review of health-related quality of life in longitudinal studies of myeloma patients. Eur J Haematol. 2017;99(1):3-17.

43. Rainville P, Carrier B, Hofbauer RK, Bushnell CM, Duncan GH. Dissociation of sensory and affective dimensions of pain using hypnotic modulation. Pain. 1999;82(2):159-171.

44. Merckaert I, Lewis F, Delevallez F, et al. Improving anxiety regulation in patients with breast cancer at the beginning of the survivorship period: A randomized clinical trial comparing the benefits of single-component and multiple-component group interventions. Psycho-Oncology. 2017;26(8):1147-1154.

45. Gregoire C, Bragard I, Jerusalem G, et al. Group interventions to reduce emotional distress and fatigue in breast cancer patients: a 9-month follow-up pragmatic trial. Br J Cancer. 2017;117(10):1442-1449.

46. Gregoire C, Faymonville M, Vanhaudenhuyse A, Jerusalem G, Willems S, Bragard I. Randomized controlled trial of a group intervention combining self-hypnosis and self-care: Secondary results on self-esteem, emotional distress and regulation, and mindfulness in post-treatment cancer patients. Quality of Life Research: An International Journal of Quality of Life Aspects of Treatment, Care & Rehabilitation. 2020:No Pagination Specified.

47. Super_Sublime. See you Sunday. https://www.supersublime.org/fr/projets/toujoursdimanche. Accessed.

Research Authorization - Translation

Integrated University Health and Social Services Centre of East Montreal Island

Quebec

April 25, 2023

Mr. David Ogez

CIUSSS of East Montreal Island

Maisonneuve-Rosemont Hospital Installation

Subject: Authorization to conduct the following research:

Project Title: Satisfaction study of an intervention integrating combined hypnosis and virtual reality procedures in the support of patients with myeloma

CEMTL assigned number: 2023-325

Mr. Ogez,

We are pleased to authorize you to conduct the research identified above in our establishment, namely the Integrated University Health and Social Services Centre of East Montreal Island

(CEMTL) - Maisonneuve-Rosemont Hospital installation.

This authorization is granted based on the documents you submitted to our establishment, notably the letter from the Research Ethics Committee (CER) of the CIUSSS of East Montreal Island dated April 25, 2023, which establishes that your research project has undergone an ethical review with a positive result and a favorable scientific review. Moreover, your project received institutional suitability approval on April 17, 2023. Should this CER inform you, during the conduct of this research, of a negative decision regarding the ethical acceptability of this research, you must consider this authorization to conduct research in our establishment revoked as of the date indicated in the CER's notice.

Our establishment has received a copy of the final version of the research documents approved by the evaluating CER.

This authorization also assumes that you commit to:

- Complying with the requests of the evaluating CER, particularly for the ethical monitoring of the research;
- Reporting to the evaluating CER and the signatory of this authorization on the progress of the project, the actions of your research team if any, and adherence to research ethics rules;
- Respecting the means related to continuous monitoring set by the evaluating CER;
- Retaining research records for the period set by the evaluating CER after the project ends, to allow

for potential verification;

- Respecting the procedures regarding the identification mechanism of research subjects in our establishment, namely: maintaining and keeping up to date the list of research subjects recruited in our establishment. This list must be provided upon request.

This authorization may be suspended or revoked by our establishment in case of non-compliance with the established conditions. The evaluating CER will be informed accordingly.

This authorization to conduct research in our establishment will be renewed annually on the date of the CER renewal.
