# Supplementary material for: Using virtual reality hypnosis during stem cell transplant for patients in hematology: A protocol for a feasibility randomized study
Source: PLoS One. 2026 Feb 27;21(2):e0338617. doi: 10.1371/journal.pone.0338617 (PMC12948061; doi:10.1371/journal.pone.0338617)
Supplement: Attachment — Submitted filename: 18_09_25_Cover letter and revision.docx [file pone.0338617.s005.docx]

**Cover letter**

Dear Editors,

Thank you for your revisions. We updated the submission. As suggested, we have resubmitted our manuscript under the Study Protocol section. Could you please confirm that it will not undergo the full peer review process again, given that it has already been reviewed by external referees?

We appreciate your clarification and look forward to your confirmation.

Best regards,

David Ogez, Pr.

Floriane Rousseaux, PhD.

**Responses to editors**

PONE-D-25-12703R1
Using Virtual Reality Hypnosis During Stem Cell Transplant for Patients in Hematology: A Protocol for a Randomized Study
Dr Floriane Rousseaux

Dear Dr. Rousseaux,

We've checked your submission and before we can proceed, we need you to address the following issues:

1. We notice that your revision was submitted on [Sep 10 2025], but the manuscript file in your submission's file inventory was uploaded on [Apr 14 2025]. Please upload the latest version of your revised manuscript as the main article file, with the item type 'Manuscript,' ensuring that it does not contain any tracked changes or highlighting. This will be used in the production process if your manuscript is accepted.

**RESPONSE** : Thank you for this observation. We removed the Apr 14 version and we add a manuscript final version in addition to the manuscript with track changes.

2. Thank you for your detailed explanation regarding the start of recruitment for your project. We regret that the peer review process has been lengthy. Unfortunately, our Registered Report Protocol format requires that recruitment begin only after the protocol is published.

Having said that, we appreciate your efforts to conduct this project with reproducibility and rigor in mind. We would be happy to consider your manuscript for publication as a Study Protocol. If you wish to have your study considered as a Study Protocol, please update the "Article Type" field in Editorial Manager accordingly. Again, my apologies for the inconvenience

**RESPONSE** : Thank you for considering our article as a Study Protocol. We updated the article type.

Best regards,

Floriane Rousseaux, PhD.
